# Independent risk factors of acute kidney injury among patients receiving extracorporeal membrane oxygenation

**DOI:** 10.1186/s12882-023-03112-6

**Published:** 2023-03-30

**Authors:** Wan Chen, Mingyu Pei, Chunxia Chen, Ruikai Zhu, Bo Wang, Lei Shi, Guozheng Qiu, Wenlong Duan, Yutao Tang, Qinwei Ji, Liwen Lv

**Affiliations:** 1 Department of Emergency, Research Center of Cardiovascular Disease, The People’s Hospital of Guangxi Zhuang Autonomous Region, Guangxi Academy of Medical Sciences, 530021 Nanning, China; 2 Department of Pharmacy, The People’s Hospital of Guangxi Zhuang Autonomous Region, 530021 Nanning, China; 3 Department of Cardiology, Research Center of Cardiovascular Disease, The People’s Hospital of Guangxi Zhuang Autonomous Region, Guangxi Academy of Medical Sciences, 530021 Nanning, China

**Keywords:** Acute kidney injury, Extracorporeal membrane oxygenation, Risk factors

## Abstract

**Objective:**

Acute kidney injury (AKI) is one of the most frequent complications in patients treated with extracorporeal membrane oxygenation (ECMO) support. The aim of this study was to investigate the risk factors of AKI in patients undergoing ECMO support.

**Methods:**

We performed a retrospective cohort study which included 84 patients treated with ECMO support at intensive care unit in the People’s Hospital of Guangxi Zhuang Autonomous Region from June 2019 to December 2020. AKI was defined as per the standard definition proposed by the Kidney Disease Improving Global Outcome (KDIGO). Independent risk factors for AKI were evaluated through multivariable logistic regression analysis with stepwise backward approach.

**Results:**

Among the 84 adult patients, 53.6% presented AKI within 48 h after initiation of ECMO support. Three independent risk factors of AKI were identified. The final logistic regression model included: left ventricular ejection fraction (LVEF) before ECMO initiation (OR, 0.80; 95% CI, 0.70–0.90), sequential organ failure assessment (SOFA) score before ECMO initiation (OR, 1.41; 95% CI, 1.16–1.71), and serum lactate at 24 h after ECMO initiation (OR, 1.27; 95% CI, 1.09–1.47). The area under receiver operating characteristics of the model was 0.879.

**Conclusion:**

Severity of underlying disease, cardiac dysfunction before ECMO initiation and the blood lactate level at 24 h after ECMO initiation were independent risk factors of AKI in patients who received ECMO support.

## Introduction

Extracorporeal membrane oxygenation (ECMO) is increasingly applied for patients complicated with cardiogenic shock as well as respiratory failure [1]. Acute kidney injury (AKI) is a common serious complication among patients treated with ECMO. The overall incidence of AKI is around 70–85% in this population. The mortality of patients developing AKI during or after ECMO is as high as 80% [2–5]. The occurrence of AKI may also be associated with a series of adverse clinical outcomes including prolonged ECMO duration, in-hospital mortality, and increased risk of chronic kidney disease (CKD) [1–4, 6].

Previous studies illustrated some risk factors of AKI, such as severity of illness and inflammatory biomarkers [7]. Hemodynamic instability could also increase the risk of AKI. In patients treated with venoarterial extracorporeal membrane oxygenation (VA-ECMO), hemodynamic instability due to cardiac dysfunction is a common feature. However, fewer current studies considered the effects of cardiac dysfunction. Therefore, it is necessary to identify the risk factors comprehensively.

The objectives of this study were to investigate the incidence of AKI and identify the risk factors in patients on ECMO.

## Materials and methods

### Study population

This is a retrospective observational cohort study conducted in the People’s Hospital of Guangxi Zhuang Autonomous Region between June 2019 to December 2020. Adult patients (> 18 years) who received ECMO support were enrolled. The Ethics Committee of the People’s Hospital of Guangxi Zhuang Autonomous Region approved this study protocol (IRB No. KY-2019-02), which abided by the Declaration of Helsinki.

### Data collection

The following clinical data were recorded: (1) demographic data, including age, sex and body mass index (BMI); (2) comorbidities including diabetes mellitus, coronary artery disease, hypertension, hepatic sclerosis and chronic obstructive pulmonary disease (COPD); (3) causes of ECMO support; (4) severity of illness including acute physiology and chronic health evaluation II score (APACHE II score) and sequential organ failure assessment score (SOFA score); (5) mode of ECMO; (6) duration of ECMO support; (7) laboratory examination result including blood urea nitrogen (BUN), serum creatinine (SCr), albumin, hemoglobin, lactate and white blood cell (WBC) count; (8) oxygenation index (OI); (9) vasoactive drug index (VIS); (10) echocardiogram data before and after initiation of ECMO support.

### Outcome and definition

The primary endpoint of this study was the occurrence of AKI within 48 h after ECMO implementation. AKI was defined as an increase in SCr by ≥ 0.3 mg/dl (≥ 26.5 µmol/l) within the first 48 h or urine volume < 0.5 ml/kg/h for 6 h after initiation of ECMO support according to the KDIGO criteria [8].

### Statistical analysis

Continuous variables that normally distributed are presented as the mean (standard deviation, SD) while non-normally distributed variables are presented as median (interquartile range, IQR). Descriptive statistic for categorical variables is presented as counts and percent frequencies. Differences between patients who did or did not develop AKI were tested by chi-square test (categorical variables) and Student’s t-test or Mann-Whitney U test (continuous variables). A preliminary investigation of the association between AKI and risk factors which were significantly different in the two groups and with missing value < 15% was conducted through univariable logistic regression analysis. Pearson correlation test was used to assess the potential interaction or collinearity between variables. To identify the independent predictors of AKI, variables with p < 0.05 in the univariate analysis were entered in a logistic regression analysis with backward stepwise selection. Multivariate analysis was conducted using 0.05 and 0.10 entry and removal probabilities, respectively. All remaining risk factors were then included in a multivariate logistic regression model. Adjusted odds ratio (OR) and 95% confidence intervals (CI) were reported as the results. Receiver operating characteristic (ROC) curves were generated to examine the performance in predicting AKI of each determined risk factor and the final fitted model. Based on the ROC curve, we calculated the area under the curve (AUC). SPSS statistical software (version 20, IBM, USA) was used for all analyses. *P* value less than 0.05 was considered statistically significant.

## Results

### Study flow chart

**Fig. 1:**
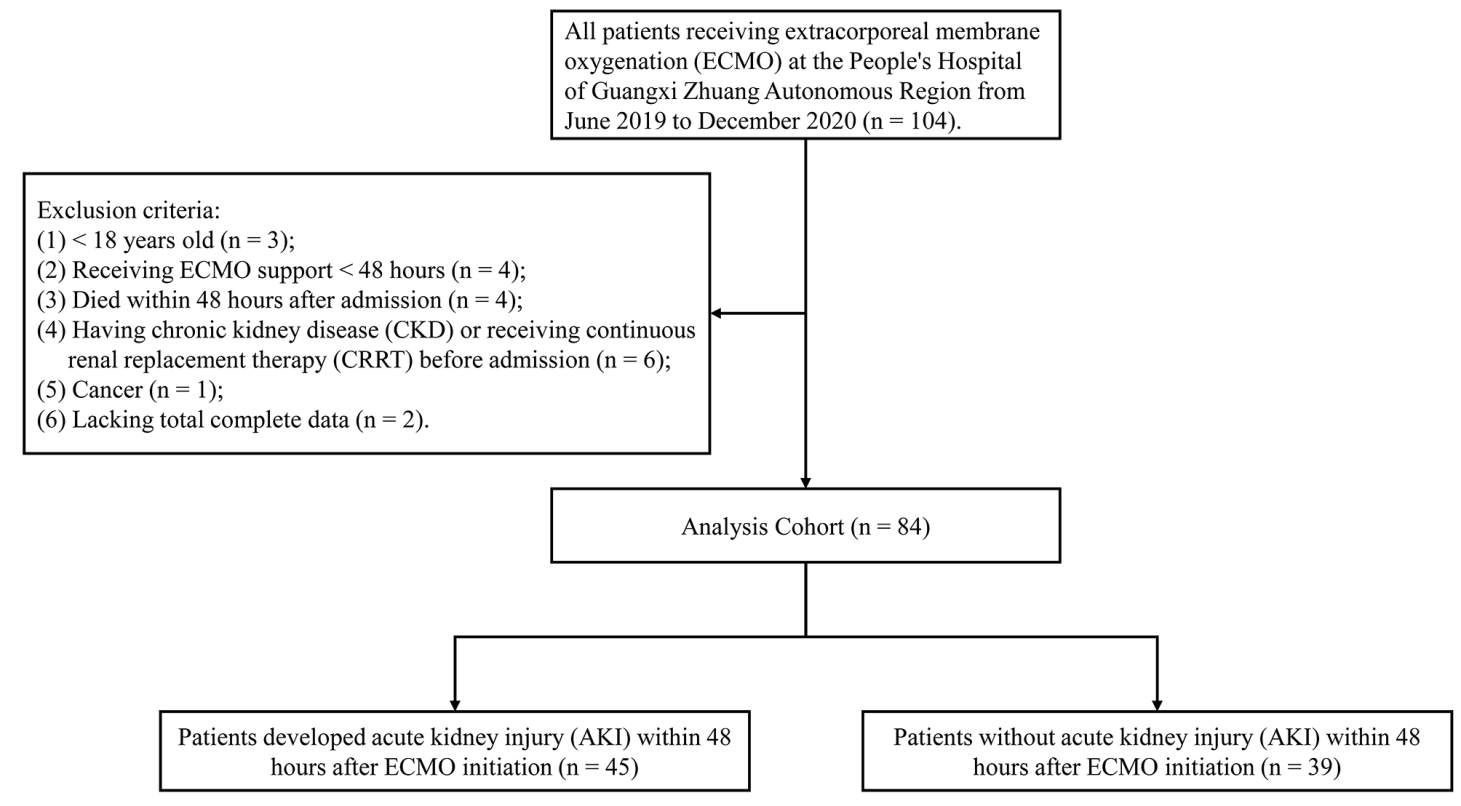
Study flow chart

The study flow chart was shown in Fig. 1. A total of 104 people were treated with ECMO at the People’s Hospital of Guangxi Zhuang Autonomous Region from June 2019 to December 2020. Patients with the following characteristics were excluded: (1) patients younger than 18 years of age (n = 3); (2) duration of ECMO less than 48 h (n = 4); (3) dying within 48 h after admission (n = 4); (4) patients with chronic kidney disease or receiving continuous renal replacement therapy before admission (n = 6); (5) cancer (n = 1); (6) lacking total complete data (n = 2). Eventually, 84 patients were selected to be enrolled in the present study.

### Characteristics of patients and incidence of AKI

A total of 84 patients with ECMO support were recruited, including 70 male (83.3%) and 14 female (16. 7%) patients, with an average age of 52.11 ± 14.21 years. The incidence of AKI was 53.57% (n = 45). Among all enrolled patients, the mean APACHE II score and the mean SOFA score were 30.77 and 12.84, respectively. The mean ECMO support time was 176.77 h and 72.6% of patients (n = 61) received the VA mode of ECMO. The mean LVEF at baseline before ECMO initiation was 36.06%. Clinical characteristics of the patient are detailed in Table 1.

**Table 1:** Basic Characteristics of the Study Population

Characteristic*	All Patients (N = 84)	AKI (n = 45)	Non-AKI (n = 39)	*P* value
**Demographic characteristics**				
Age, years	52.11 (14.21)	52.24 (13.43)	51.97 (15.23)	0.93
Male, n (%)	70 (83.3)	38 (84.4)	32 (82.1)	0.78
Female, n (%)	14 (16.7)	7 (15.6)	7 (18.00)	0.78
BMI, (kg/m^2^)	23.17 (2.69)	23.50 (2.68)	22.68 (2.65)	0.13
**Severity of illness**				
APACHE II score	30.77 (10.25)	35.26 (9.36)	25.58 (8.75)	< 0.001
SOFA score	12.84 (3.78)	14.62 (3.23)	10.79 (3.32)	< 0.001
**Causes of ECMO support**				
ARDS, n (%)	16 (19.1)	5 (11.1)	11 (28.2)	0.04
Non-ARDS lung causes, n (%)	7 (8.3)	0 (0.0)	7 (18.0)	0.003
Post-cardiotomy complications, n (%)	12 (14.3)	5 (11.1)	7 (18.0)	0.37
Non-operative cardiac causes, n (%)	35 (41.7)	24 (53.3)	11 (28.2)	0.02
Others, n (%)	13 (15.5)	5 (11.1)	8 (20.5)	0.24
**Comorbidities**				
COPD, n (%)	8 (9.2)	3 (6.7)	5 (12.8)	0.46
Hypertension, n (%)	9 (10.3)	6 (13.3)	3 (7.7)	0.49
Type 2 diabetes mellitus, n (%)	7 (8.1)	5 (11.1)	2 (5.1)	0.44
Coronary artery disease, n (%)	5 (5.8)	4 (8.9)	1 (2.6)	0.37
hepatic sclerosis, n (%)	5 (5.8)	1 (2.2)	4 (10.3)	0.37
**Laboratory examinations**				
WBC count, 10^9^/L	14.93 (8.06)	15.02 (8.41)	14.97 (7.63)	0.98
Hemoglobin, g/L	95.83 (26.45)	92.13 (21.98)	100.10 (30.56)	0.17
Albumin, g/L	30.77 (1.77)	31.04 (1.64)	30.57 (1.91)	0.23
CRP, mg/dL	42.08 (14.08)	42.88 (13.23)	41.16 (15.13)	0.58
Platelet count, 10^9^/L	125.95 (16.26)	127.38 (16.06)	124.31 (16.55)	0.39
Creatinine, µmol/L				
before ECMO initiation	81.0 [71.0–87.0]	82.0 [72.0–87.0]	80.0 [70.0-86.5]	0.71
24 h after ECMO initiation	145.5 [29.0-703.0]	220.0 [74.0-703.0]	84.0 [29.0-149.0]	< 0.001
48 h after ECMO initiation	100.0 [25.0-475.0]	159.0 [57.0-47.05]	74.0 [25.0-121.0]	< 0.001
BUN, mmol/L				
24 h after ECMO initiation	11.8 [3.5–38.6]	16.6 [4.6–38.6]	8.5 [3.5–15.2]	< 0.001
48 h after ECMO initiation	8.4 [1.7–38.2]	9.8 [3.2–38.2]	7.3 [1.7–18.7]	0.007
Lactate, mmol/L				
before ECMO initiation	6.8 [2.5, 14.1]	7.9 [5.2–14.9]	4.0 [1.9–9.7]	0.002
24 h after ECMO initiation	4.7 [2.50–10.8]	8.50 [3.2–13.6]	3.2 [2.2–6.6]	0.001
48 h after ECMO initiation	2.20 [1.5–3.7]	2.60 [1.5–5.7]	1.80 [1.5–2.8]	0.01
VIS count				
before ECMO initiation	54.0 [30–140.0]	70.0 [40.0-165.0]	45.0 [9.5-102.5]	0.008
24 h after ECMO initiation	101.57 (309.10)	116.26 (363.78)	84.62 (234.32)	0.64
48 h after ECMO initiation	134.29 (471.15)	214.02 (626.80)	42.31 (117.16)	0.08
Urine volume, mL				
0 to 24 h after ECMO initiation	1575.0 [433.5–2780.0]	556.0 [75.0-2375.0]	2265.0 [1430.0-2885.0]	< 0.001
24 to 48 h after ECMO initiation	1385.0 [434.2-2862.5]	590.0 [40.0- 2055.0]	2390.0 [1330.0, 3287.5]	< 0.001
MAP, mmHg				
before ECMO initiation	31.68 (12.08)	32.59 (12.08)	30.63 (12.16)	0.46
24 h after ECMO initiation	81.56 (17.27)	81.19 (17.61)	81.98 (17.08)	0.84
48 h after ECMO initiation	81.39 (17.31)	81.89 (18.20)	80.82 (16.45)	0.78
OI, mmH_2_O				
before ECMO initiation	88.52 (39.96)	84.75 (35.34)	92.87 (44.77)	0.36
24 h after ECMO initiation	329.67 (190.58)	352.64 (210.62)	303.17 (163.14)	0.24
48 h after ECMO initiation	289.75 (140.38)	293.71 (147.86)	285.17 (133.00)	0.78
**Echocardiography**				
LVEF, %				
before ECMO initiation	36.06 (17.08)	29.85 (16.83)	43.23 (14.51)	< 0.001
24 h after ECMO initiation	48.92 (11.09)	46.15 (11.58)	52.12 (9.68)	0.01
48 h after ECMO initiation	52.63 (12.21)	50.00 (12.51)	55.67 (11.25)	0.03
**Medicine**				
Nephrotoxic drug (n, %)	26 (29.9)	17 (37. 8)	9 (21.4)	0.10
Diuretic (n, %)	8 (9.2)	5 (11.1)	3 (7.1)	0.48
**Procedure**				
ECMO support time, h	176.77 (130.82)	167.46 (114.52)	187.51 (148.24)	0.49
VA-ECMO mode (n, %)	61 (72.6)	40 (88.9)	21 (53.9)	< 0.001
ICU hospitalization time, day	15.0 [8.0-22.2]	13.0 [7.0–22.0]	18.0 [10.0–28.0]	0.02
Total length of hospital stays, day	27.09 (18.83)	24.93 (20.94)	29.58 (18.40)	0.29

* Data are presented as the mean value (standard deviation), median [interquartile range] or number of participants (percentage)

Abbreviations: AKI, acute kidney injury; BMI, body mass index; APACHE II score, acute physiology and chronic health evaluation II score; SOFA score, sequential organ failure assessment score; ECMO, extracorporeal membrane oxygenation; ARDS, acute respiratory distress syndrome; COPD, chronic obstructive pulmonary disease; WBC, white blood cell; CRP, C-reactive protein; BUN, blood urea nitrogen; LVEF, left ventricular ejection fraction; VIS, vasoactive inotropic score; MAP, mean arterial pressure; OI, oxygenation index; VA-ECMO, veno-arterial extracorporeal membrane oxygenation ; ICU, intensive care unit

Among 45 patients with AKI, all patients met the diagnostic criteria for AKI with an absolute creatinine increase greater than 0.3 mg/dl (26.5 µmol/l) within the first 48 h. Meanwhile, 26 patients had both an absolute creatinine increase greater than 0.3 mg/dl (26.5 µmol/l) within the first 48 h and a urine output of less than 0.5 ml/kg/h for 6 h after initiation of ECMO.

### Risk factors for AKI

According to the result of multivariate logistic regression analysis with backward stepwise, LVEF before ECMO initiation (OR, 0.80; 95% CI, 0.70–0.90; *P* < 0.001), SOFA score before ECMO initiation (OR, 1.41; 95% CI, 1.16–1.71; *P* = 0.001), and serum lactate at 24 h after ECMO initiation (OR, 1.27; 95% CI, 1.09–1.47; *P* = 0.002) were independently associated with AKI occurrence during hospitalization. Noteworthily, higher LVEF is a protective factor for AKI, as a 1% increase in LVEF reduces the risk of AKI by 20% (Table 2).

**Table 2:** Risk factors of post-ECMO AKI determined by multivariate analysis with backward stepwise

	Univariate				Multivariate		
	OR	95% CI	P Value		OR	95% CI	P Value
Age	1.00	0.97–1.03	0.93				
Male	1.19	0.38–3.74	0.77				
APACHE II score	1.13	1.06–1.20	< 0.001				
SOFA score	1.47	1.22–1.77	< 0.001		1.41	1.16–1.71	0.001
Creatinine before ECMO initiation	1.00	0.98–1.03	0.91				
Creatinine at 24 h after ECMO initiation	1.07	1.04–1.11	< 0.001				
Creatinine at 48 h after ECMO initiation	1.06	1.03–1.09	< 0.001				
BUN at 24 h after ECMO initiation	1.33	1.17–1.52	< 0.001				
BUN at 48 h after ECMO initiation	1.16	1.04–1.30	0.006				
Lactate before ECMO initiation	1.13	1.04–1.23	0.004				
Lactate at 24 h after ECMO initiation	1.20	1.08–1.33	0.001		1.27	1.09–1.47	0.002
Lactate at 48 h after ECMO initiation	1.49	1.10–2.03	0.01				
LVEF before ECMO initiation	0.94	0.91–0.98	0.001		0.80	0.70–0.90	< 0.001
LVEF at 24 h after ECMO initiation	0.94	0.90–0.99	0.02				
LVEF at 48 h after ECMO initiation	0.96	0.92-1.00	0.04				

Abbreviations: LVEF, left ventricular ejection fraction; APACHE II score, acute physiology and chronic health evaluation II score; SOFA score, sequential organ failure assessment score; ECMO, extracorporeal membrane oxygenation

### Correlations between risk factors determined by stepwise regression and baseline creatinine level

**Fig. 2:**
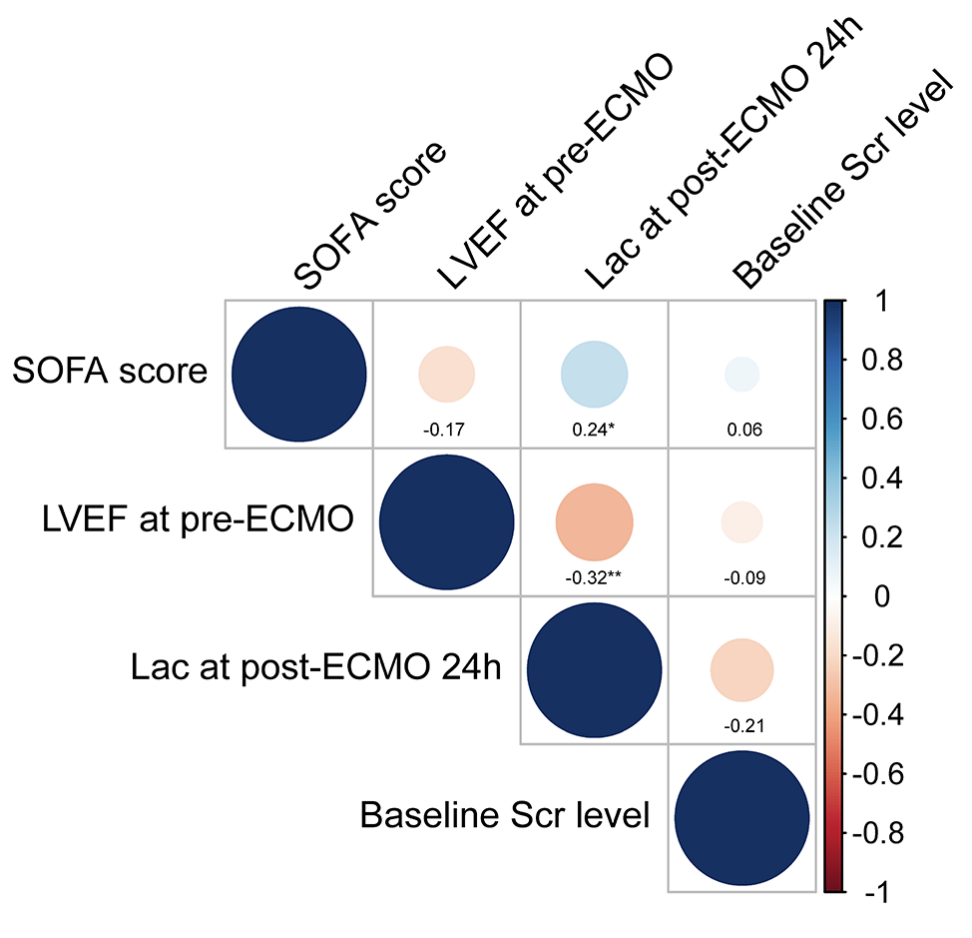
Correlations between risk factors determined by stepwise regression and baseline creatinine level. *p-value for spearman correlation < 0.05, **p-value for spearman correlation < 0.01

Figure 2 showed all correlations between the 3 determined risk factors and baseline serum creatinine concentration. Lactate at 24 h after ECMO initiation was weakly though significantly correlated with SOFA score before ECMO initiation (r = 0.24, *P* = 0.03) and pre-ECMO LVEF (r = -0.32, *P* = 0.003). SOFA score before ECMO initiation showed no significant correlation with LVEF before ECMO initiation (r = -0.17, *P* = 0.13). All the 3 risk factors were not related to baseline serum creatinine (all *P* > 0.05).

### Performance of the model in prediction of AKI development

**Fig. 3:**
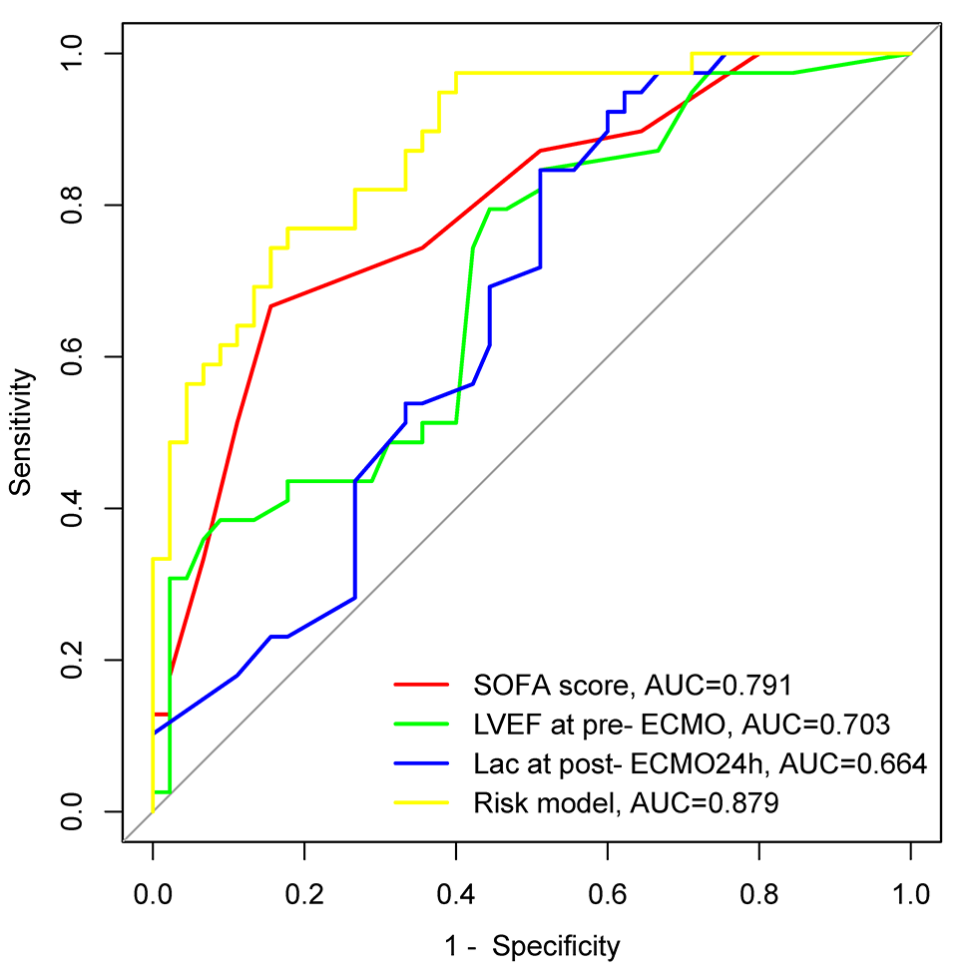
Receiver operating characteristic curve of the logistic regression model constructed to predict AKI. The area under the curve (AUC) and 95% confidence interval (CI) values are given inside the ROC curve

As shown with the ROC curve, the fitted risk model was effective in predicting the development of AKI (AUC, 0.879; 95% CI, 0.809–0.950). A sensitivity of 0.933 and a specificity of 0.667 were assessed (Fig. 3).

## Discussion

This study described the characteristic of patients treated with ECMO, demonstrated the incidence of AKI among this population and identified the risk factors of AKI comprehensively. The incidence of AKI was 53.6% among these patients according to the present study. SOFA score, LVEF before ECMO initiation, and lactate at 24 h after ECMO initiation were independent risk factors of AKI. The three risk factors all had good performance in predicting AKI.

Among patients receiving ECMO treatment, AKI is a common and severe complication that influences prognosis [2, 6, 9–13]. Our study showed that the overall incidence of AKI in patients undergoing ECMO was 53.6%, which is similar to that reported in a previous study [14]. In the present study, we also found a 58.1% in-hospital mortality, which is similar to 42.9–70.2% mortality rates reported by previous multicenter cohort studies [14, 15]. The mortality and incidence in our study may vary due to the severity of the etiology, the different evaluation criteria for AKI, and the different types of diseases.

In our study, we found that an elevated preoperative SOFA score was associated with an increased risk of AKI which occurred after ECMO initiation. SOFA score, measuring multiple organ failure, is positively correlated with the illness severity. Patients in AKI group had higher SOFA scores, indicating severe status of organ failure. In previous studies, both SOFA and APACHE II scores predicted organ insufficiency and prognosis in critically ill patients. Moreover, the SOFA score showed a better predictive performance than that of the APACHE II score in multiple diseases [16]. In Ding et al.’s study, it was demonstrated that SOFA score was associated with persistent AKI in patients admitted to ICU according to the Medical Information Mart for Intensive Care IV (MIMIC-IV) database [17]. In Zhang et al.’s study, it was also demonstrated that SOFA score was independently associated with the new development of AKI among 2525 patients admitted to a multi-disciplinary ICU in a single tertiary care center in the UK [18]. Notwithstanding the lack of correlation between SOFA score and plasma creatinine value before ECMO initiation, as renal function is a component of the SOFA score, it is possible that the patients with higher SOFA score at admission also had worse renal function at baseline.

LVEF before ECMO initiation was an independent protective factor of AKI among patients treated with ECMO and every 1% increase in LVEF value caused a 20% decrease in the risk of AKI (OR: 0.80, 95% CI: 0.70–0.90, *P* < 0.001). Lower LVEF values before ECMO were suggestive of more severe cardiac failure and shock before initiation of ECMO support therapy, and decreased cardiac output and low arterial perfusion pressure often implied a worse perfusion status. In a multicenter study of 6,112 patients with acute heart failure [19], lower LVEF was associated with more pronounced deterioration of the renal function during hospitalization and strongly related to the occurrence of adverse cardiovascular events after discharge (OR: 1.2, 95% CI: 1.00-1.43, *P* = 0.050). Another single-center study on a myocardial infarction population noted that more severe myocardial infarct size (OR: 3.02, 95% CI: 1.85–4.93) was associated with a high risk of AKI [20].

The multivariable regression analysis result also indicated that blood lactate values at 24 h after ECMO initiation was an independent risk factor of AKI among the study population. Blood lactate is the end product of glycolysis, and is a reflection of the state of the body’s microcirculation, especially the presence of microcirculatory disturbances in the kidneys in pathological states [21, 22]. However, abnormally elevated blood lactate level signifies that the body is already in a state of severe hypoxia and represents the body’s positively or negatively circulatory status. In a single-center study of 2,331 critically ill patients heart failure (OR: 2.30) and lactic acidosis (OR: 2.16) were independent risk factors for AKI [23]. Another observational study also demonstrated that a high peak lactate level (OR: 1.44) was independently associated with AKI in patients undergoing elective coronary artery bypass graft surgery [24].

Together these findings suggest that in patients on ECMO, renal perfusion before/after treatment plays a decisive role in the development of AKI. Although the kidneys receive approximately 20% of the cardiac output and have low fractional oxygen extraction, they are highly susceptible to ischemic injury that is most evident in the renal medulla [25]. There are several possible mechanisms that could explain this phenomenon. First, as concluded by Brezis et al. [26], oxygen tension is low under ischemic conditions owing to the high metabolic demand (high transport) of tubular cell energy metabolism disorder and continued tubular transport; this process could be the cause of ischemic injury in tubular cells. The second theory is based on the fact that the outer medullary capillary network is extremely vulnerable to vascular obstruction during periods of low perfusion. Congestion of the microvasculature then prevents reperfusion after the initial ischemic event has passed, thereby prolonging the ischemic event [27–29].

AKI has a significant impact on patient prognosis in previous studies. In the present study, statistical difference significantly existed among patients with and without AKI (*P* = 0.02). In multivariate analysis, lactate at 24 h after ECMO initiation was the only factor affecting the prognosis of ECMO patients (OR, 1.27; 95% CI, 1.09–1.47; *P* = 0.002). Our results are at variance with those of Lee et al. [30], conceivably due to different patients’ characteristics, reasons for ECMO support, and time of AKI onset since admission. Moreover, in our study, most patients needed ECMO support treatment because of cardiogenic shock. For such patients, early blood perfusion and oxygen recovery are essential to save the lives of patients[31].

Another phenomenon should also be taken seriously. As shown in Table 1, the concentration of creatinine in 24 h after ECMO initiation was the highest. Creatinine levels at 48 h after ECMO initiation were significantly lower than those at 24 h after ECMO initiation. As for the urine output, its median value increased numerically, albeit not significantly due to small sample size, at the second compared to the first 24-h period after ECMO initiation. Since our study enrolled patients receiving ECMO, the most prominent feature of these patients was hemodynamic instability. It is precise because hemodynamic instability can directly lead to renal hypoperfusion and microcirculation disturbance, which leads to the occurrence of acute kidney injury. ECMO support can quickly correct the state of hemodynamic instability. It should be noted that AKI was caused by the severe condition of the patient accompanied by hemodynamic instability. After the initiation of ECMO support, the hemodynamic instability was corrected and renal perfusion improved.

Some limitations should also be considered in this study. As this is a retrospective study, insufficient data may have led to underestimation of the prevalence of risk factors. Second, due to the rapid progression of the disease and the pressing time for rescue, collected data are limited on study population. Because of the retrospective design of the study, notwithstanding multivariable analysis residual confounding is likely. Third, some patients needed CRRT treatment after AKI, which may affect our judgment on the classification of AKI.

## Conclusion

In conclusion, pre-ECMO SOFA score, LVEF before ECMO initiation, and lactate level at 24 h after ECMO implementation were independent risk factors of AKI in the early phase among patients receiving ECMO treatment. Severe preoperative organ dysfunction, hemodynamic instability caused by decreased cardiac output before ECMO initiation and microcirculation hypoxia at early stage after ECMO initiation increase the risk of AKI development.

## Data Availability

Not applicable at this stage. The datasets analyzed during the current study will be available from the corresponding author upon reasonable request.
